# The Palliative Treatment of Pulmonary Tuberculosis

**Published:** 1894-10

**Authors:** C. M. Boger

**Affiliations:** Parkersburg, West Va.


### THE PALLIATIVE TREATMENT OF PULMONARY TUBERCULOSIS.

C. M. Boger, M. D., Parkersburg, West Va.

The treatment of pulmonary tuberculosis resolves itself into two general methods, the curative and the palliative, the former ideal in its conception aims to eradicate the fundamental causative miasm, the latter by its very existence concedes the improbability of attaining this end and endeavors to procure euthanasia for the sufferer.

From the Hahnemannian standpoint there can be only one treatment, and that the indicated remedy. That this is so often unsatisfactory or even disastrous in its results, is, I think, due to the fact that the prescription is the simillimum to the original or causative disease-producing miasm. It is removing the foundation stones first, when taking down the disease edifice, and the consequences are naturally disastrous. According to the principles of sound logic, the precepts of *The Organon*, and above all actual experience, the reverse process must be applied if we expect to cure; the superstructure must be taken down in regular order and the foundation taken up last of all. By this I do not mean to say that the cure of this disease lies through palliation, although the palliator will often see wonderful results. Take for instance those cases which we all see, after the formation of vomicæ and through them the resorption of toxic poisons producing symptoms simulating the zymoses. The very first step here must be the removal of this apparent zymotic disease picture, for is it not really another miasm implanted by means of these toxines on one already present? Whether its symptoms resemble intermittent, remittent, or even the hectic of suppuration; although as a matter of fact they most frequently simulate the former, this naturally does not rule out the fact that a pure fever may, although it rarely does, complicate phthisis. Until this zymotic state is removed not a single step of progress toward a cure can be made, nay the case cannot even be fairly palliated.

The remedies useful in these cases are those which correspond to respectively similar febrile disease states, and although any remedy may be indicated China easily leads all others, especially in cases coming from our allopathic friends which have been dosed fully on Quinine. Its other indications are well known. In the 1M potency it has for me removed in a few days, symptoms which had for months resisted old-school treatment.

The next most frequently indicated remedies in the order of their importance here according to my experience are Nat-m., Nux-v., Kali-c., Lyc., and Polyporus. When the similar is chosen the chill, heat, and sweat disappear as if by magic, the expectoration is temporarily augmented but then quickly declines and loses its muco-purulent character, and, even in hopeless cases, general all-around improvement sets in. Physical diagnosis aside, the general aspect of the case and the next remedy which the symptoms now call for will constitute valuable factors in the prognosis, and right here is the place to put an empirical prescription, although generally speaking this is a thing to be avoided. My custom is to give one dose Tuberculinum$^{dmm}$ (Swan), especially if the patient be a blonde of the sanguine temperament, or Calc-phos.$^{1m}$, if a brunette with a predominating venous system. From these two remedies in just this place I have seen the happiest results, and they will do what few others will—*i. e.*, establish reaction in cases where this seems absent, and following this the symptoms will usually unfold beautifully, indicating the appropriate remedy.

If the night-sweats become troublesome Calcar-carb.$^{200}$ deserves the first place, although Kali-c.$^{3c}$, Polyporus$^{3x}$, Atropine$^{3x}$, or any other remedy may be indicated. I would especially call attention to Polpyorus-off. in cases presenting a combination of hepatic and intermittent symptoms often coupled with night-sweats. In this sphere it is almost unique and certainly out-ranks all remedies, with its botanical relative Agaricus next and perhaps Lachesis third.

Another type of cases are marked by a hemorrhagic tendency, usually taking the form of hæmoptysis. For this the remedies are as various as the symptoms. I will mention only those more generally indicated in cases coming under my observation:

*Geranium-maculat.Φ*, is here the prince of remedies and is prescribed empirically. I have never prescribed it without some benefit even when it did not totally remove this symptom. It appears to be more efficacious where the evidence of inflammation is unmistakable.

*Ferrum-phos.*, when passive congestion and chlorosis are coexistent.

*Laurocerasus* with deficient reaction there is persistent blood-flecked expectoration.

Millefol. and Ipecac. deserve mention.

Still another class of cases present many gastro-intestinal symptoms, the most serious of which are vomiting following the cough paroxysm and diarrhœa. It is important that the former be promptly met, and our repertories give a number of remedies of which Coccus-cacti. is easily the best, especially where the vomiting is excited by irritation of the posterior pharynx, uvula, or epiglottal region whether from the presence of adherent expectoration (like Kali-bi.), or the simple act of coughing. This remedy has a number of times removed this symptom for me; twice it has cured non-phthisical patients of vomiting excited by the act of swallowing. Both said that as soon as the water reached the back of the mouth vomiting at once ensued.

This brings us to the diarrhœa of phthisis, and the physician who can even palliate it is deserving of being called a good prescriber. Many remedies have been urged as specifics for this condition, but the very nature of the affection makes its permanent removal more than problematical. Phos., Pod., Puls., Petrol., etc., have been extolled for this symptom, but even when seemingly indicated I have yet to see the first lasting amelioration from their use.

For undigested stools China leads all others, and may be useful. When cheezy masses are present *Calc-phos.* is more serviceable than Iod. or Thuja.

With black watery stools having the odor of rotten eggs with urinary symptoms, Asclepias-tuberos. has done magnificent work for me. It should be compared with Psorinum.

Respiratory spasm during phthisis may become prominent enough to require separate attention. Here Antimon-arsenicosum and Asthmatos-cil. (of Swan) stand in the front rank. The former is most useful in the last stages when paralysis threatens from accumulated products with a well-marked spasmodic element. Its nearest relatives here are Antimon-tart. and Grindelia-robusta, the latter an exceedingly valuable remedy when the patient stops breathing on falling asleep (like Curare, Gels., or Lach.).

Asthmatos-cil. is one of Swan's nosodes, and is without a rival in pure respiratory spasm as we see it in asthma, although it does unequaled work in consumption also.

*Thuja* should not be overlooked. In cases resting on a sycotic basis it will do what no other medicament can. I have kept a patient alive for more than four years on this remedy, and she is in better health to-day than ever. She has received in single doses the following successive potencies, 30, 200, 1M, CM, and MM.

*Aralia-racemosa,* the spikenard of our mothers, is valuable when the patient is awakened by the cough after having slept some time, and should frequently be prescribed when Lachesis is.

*Spongia* fills a niche where glandular enlargement and laryngeal symptoms correspond.

Nux-vom., Hep., and Puls. are important as indicated.

For the headaches of these sufferers Bell. is often given, but Tuberculin (DMM only) will do much better work. Try it.

